# A Novel CFEM Effector in *Fusarium verticillioides* Required for Virulence Involved in Plant Immunity Suppression and Fungal Cell Wall Integrity

**DOI:** 10.3390/ijms26094369

**Published:** 2025-05-04

**Authors:** Huan Li, Shumila Ishfaq, Xiaoyan Liang, Rui Wang, Hailei Wei, Wei Guo

**Affiliations:** 1 Institute of Food Science and Technology, Chinese Academy of Agricultural Sciences/Key Laboratory of Agro-Products Quality and Safety Control in Storage and Transport Process, Ministry of Agriculture and Rural Affairs, Beijing 100193, China; lihuancaas@126.com (H.L.); 2020y90100028@caas.cn (S.I.); liangxy_97@163.com (X.L.); wangrui_0121@126.com (R.W.); 2 Institute of Agricultural Resources and Regional Planning, Chinese Academy of Agricultural Sciences, Beijing 100081, China; weihailei@caas.cn; 3 Ningxia Key Laboratory for the Development and Application of Microbial Resources in Extreme Environments, North Minzu University, Yinchuan 750030, China

**Keywords:** *Fusarium verticillioides*, effectors, CFEM domain, pathogenicity, host–pathogen interaction, maize

## Abstract

Common in Fungal Extracellular Membrane (CFEM) effectors, a unique class of fungal-specific proteins, play critical roles in host-pathogen interactions. While CFEM proteins have been extensively characterized in phytopathogens, their presence and functions in *Fusarium verticillioides* remained unexplored. Here, we systematically identified 19 CFEM-containing proteins in *F. verticillioides*, among which FvCFEM12 exhibited secretory activity and plant infection-induced expression. Functional characterization revealed that FvCFEM12 suppressed Bax- and INF1-triggered cell death in *Nicotiana benthamiana* leaves. Furthermore, heterologous expression of FvCFEM12 in maize leaves using *P. syringae* strain D36E can compromise immune responses against bacterial pathogens. Deletion of FvCFEM12 impaired fungal virulence, altered hyphal morphology, and reduced cell wall stress tolerance. Interestingly, FvCFEM12 physically interacted with the maize wall-associated receptor kinase ZmWAK17ET, and targeted silencing of ZmWAK17 in maize enhanced susceptibility to *F. verticillioides*. Our findings revealed that FvCFEM12 is a dual-function effector that suppresses plant immunity and maintains fungal cell wall integrity, thereby orchestrating fungal pathogenicity at the host–pathogen interface.

## 1. Introduction

Plants have evolved a multitude of immune mechanisms against pathogen infection and colonization during their long-term interactions with pathogens [1]. The plant immune system primarily involves two distinct layers of immunity: pathogen or microbe-associated molecular pattern (PAMP/MAMP)-triggered immunity (PTI) and effector-triggered immunity (ETI) [1,2]. Evolutionarily conserved PAMPs such as bacterial flagellin, elongation factors, and fungal chitin are evolutionarily conserved and recognized by plant transmembrane pattern recognition receptors (PRRs) and trigger PTI. The PTI response typically triggers a cascade of cellular and physiological responses, including reactive oxygen species (ROS) burst, callose deposition, and regulation of defense-related genes [3]. To counteract host defenses, pathogens often secrete effectors to interfere or disrupt the host PTI response. However, some effectors may be recognized by intracellular nucleotide binding–leucine rich repeat receptors (NB-LRRs) and induce a more robust and rapid defense response known as ETI. ETI usually results in a hypersensitive response (HR) at the infection sites [4]. Despite these defense mechanisms, adapted pathogens can sometimes evade and overcome both PTI and ETI, ultimately leading to successful infection [5].

*Fusarium verticillioides* is a globally devastating maize pathogen, causing severe ear rot and stalk rot diseases that result in substantial yield losses worldwide [6]. It can infect all parts of maize plants including seeds, roots, leaves, stalks, and ears at every growth stage. *F. verticillioides* can survive in crop residues and soil, serving as the primary inoculum source in natural infections [7,8]. In addition to its detrimental impact on crop productivity, *F. verticillioides* is notorious for producing toxic secondary metabolites, particularly fumonisins, which pose serious health risks to both animals and humans. Among these, Fumonisin B_1_ (FB_1_) is the most prevalent and hazardous, recognized globally as a major food and feed contaminant with documented systemic toxicity and potential carcinogenicity [6,9,10]. As a hemibiotrophic fungal pathogen, *F. verticillioides* acquires nutrients from both living and dead tissues [11]. At the initial biotrophic phase, hemibiotrophic pathogens usually synthesize and secrete effector proteins to evade or suppress host immune responses [12,13]. The genome of *F. verticillioides* is predicted to encode numerous effector proteins, including cell wall-degrading enzymes, necrosis-inducing proteins, and small cysteine-rich proteins [14]. However, the specific roles of many of these effectors remain elusive. Notably, the secreted protein FvLcp1, containing a LysM domain, has been shown to suppress BAX-triggered plant cell death in *N. benthamiana* [15]. Additionally, a metalloprotease secreted by *F. verticillioides* can cleave maize class IV chitinases, which are involved in plant defense against fungal pathogens [16].

The CFEM (Common in Fungal Extracellular Membrane) protein has only been identified in fungi to date, characterized by eight cysteine residues that are equally spaced (PxC[A/G]x2Cx8-12Cx1-3[x/T]Dx2-5CxCx9-14Cx3-4Cx15-16C), where x can be any amino acid [17]. CFEM proteins exhibit a significant functional diversity across fungal species [18,19]. For instance, in *Saccharomyces cerevisiae*, the CCW14 protein is essential for maintaining cell wall integrity, while in *Aspergillus fumigatus*, CfmA-C proteins play a crucial role in maintaining the integrity and stability of the cell wall [20]. In the human pathogenic fungus *Candida albicans*, three CFEM proteins, Rbt5, Pga7, and Csa2, are part of a system responsible for iron acquisition from heme and hemoglobin [21]. Recently, the CFEM proteins have gained increased attention for their roles in plant–pathogen interactions. There are two types of CFEM proteins: Pth11-like proteins, which contain multiple transmembrane domains, and non-Pth11-like proteins, which are secreted [17]. For example, the CFEM protein Pth11, with seven transmembrane regions, is critical for appressorium formation in *Magnaporthe oryzae*, a specialized structure essential for host tissue penetration [22]. Moreover, the CFEM protein WISH is involved in mycelial growth, cell wall integrity, appressoria development, and virulence in *M. oryzae* [23]. Secreted CFEM proteins have also been identified as a potential effector in some plant pathogenic fungi [24,25,26]. For instance, BcCFEM1 in *Botrytis cinerea*, VdSCP76/77 in *Verticillium dahliae*, and PstCFEM1 in *Puccinia striiformis* f. sp. tritici have been reported to contribute to virulence, although their precise mechanisms remain poorly understood [27,28,29]. Notably, in *Colletotrichum fructicola*, the CFEM containing protein CfEC12 binds NPR1 regulator NIMIN2 to suppress plant immunity [30]. Despite these findings, the roles of CFEM proteins in *F. verticillioides* are largely unexplored, and their mechanisms of action remain largely unknown.

In this study, we identified 19 CFEM proteins in the *F. verticillioides* genome, seven of which were demonstrated to be secreted and capable of suppressing BAX-induced cell death. Among these, FvCFEM12 was found to play a critical role in disrupting plant immune responses and contributing to fungal virulence. Furthermore, FvCFEM12 was found to be involved in regulating fungal colony morphology and cell wall synthesis. Finally, to investigate the molecular mechanisms underlying FvCFEM12’s function, we identified an interaction between FvCFEM12 and the maize wall-associated kinase ZmWAK17ET. Collectively, our findings were to identify the CFEM protein in *F. verticillioides* and elucidate the molecular function of FvCFEM12 as a candidate effector in fungal physiology and pathogenicity.

## 2. Results

### 2.1. Bioinformatic Identification of CFEM Proteins in F. verticillioides

**Figure 1:**
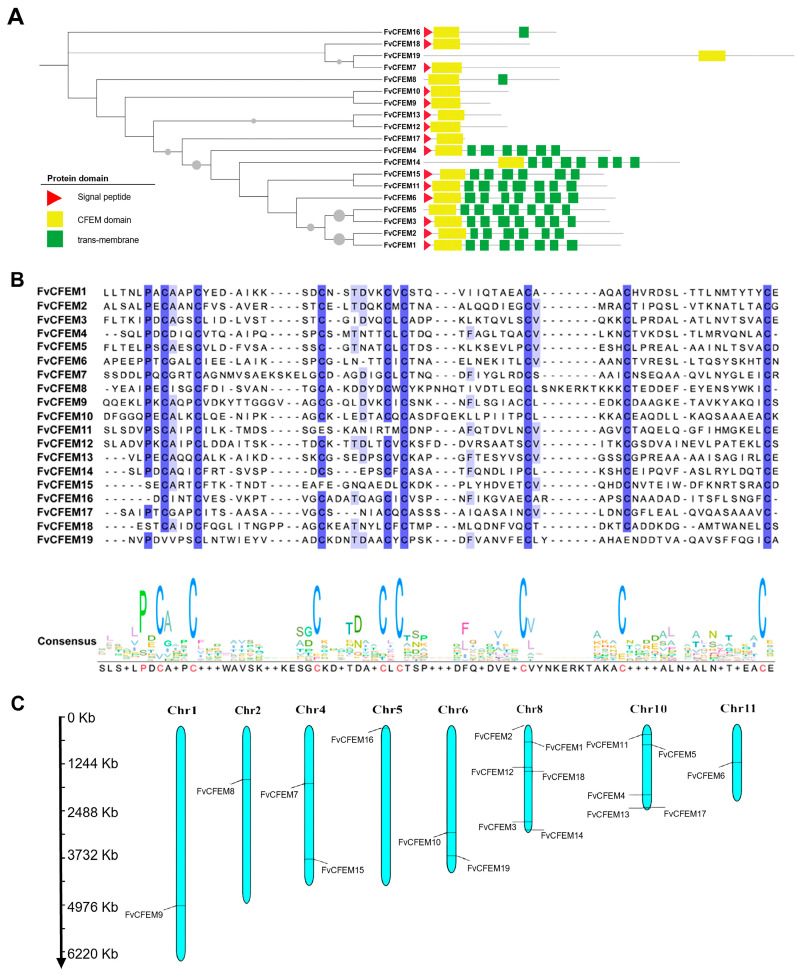
Bioinformatic analysis of Common in Fungal Extracellular Membrane (CFEM) proteins in *F. verticillioides*. (**A**) A neighbor-joining phylogenetic tree was constructed based on the amino acid sequences of FvCFEM proteins. Branches with bootstrap values > 70% are shown, with the size of grey spheres indicating bootstrap values. Protein domain architectures of FvCFEMs are depicted on the right, with different colors representing distinct domains: red triangles for signal peptides (SPs), yellow boxes for CFEM domains and green boxes for transmembrane (TM). (**B**) Multiple sequence alignment of FvCFEM proteins. The alignment was performed using ClustalW 1.1 and visualized using Jalview 2.11.3.2. (**C**) Mapping of the CFEM genes on *F*. *verticillioides* chromosomes.

A total of 19 CFEM proteins (designated FvCFEM1-FvCFEM19) were identified in the *F. verticillioides* genome using BLASTp analysis, using ACI1(AAN64312.1) from *M. oryzae* as the query sequence [31]. Subsequent SMART domain analysis using the SMART tool confirmed the presence of a single CFEM domain in all identified proteins. The protein ID number and associated features retrieved from the NCBI database are shown in Table 1 and Figure 1A, respectively. The predicted length of the CFEM-domain proteins ranged from 95 to 1018 amino acids. Among these, seven proteins were predicted to contain an N-terminal signal peptide, indicating their potential secretory function.

A multiple sequence alignment of the CFEM domains was performed using the amino acid sequences of the identified proteins (Figure 1B). The alignment revealed a high degree of conservation within the CFEM domain across all sequences, with the domain length consistently ranging from 58 to 71 amino acids. Except FvCFEM 11, 14, 15, 16, 18, and 19, most of the CFEM proteins contained eight cysteine residues, which are probably involved in the formation of disulfide bonds to stabilize the whole protein structure of CFEM domain [21].

To elucidate the chromosomal distribution of putative 19 CFEM genes, we performed genome-wide mapping of *F. verticillioides* using MG2C v2.1 on the website (http://mg2c.iask.in/mg2c_v2.1/index.html, accessed on 20 July 2024). The 19 identified FvCFEM genes were distributed across eight chromosomes, displaying an uneven distribution pattern. Notably, chromosome 8 harbored the highest number of FvCFEM genes (*n* = 6), while chromosomes 1, 2, 5 and 10 each contained only a single FvCFEM gene (Figure 1C).

### 2.2. FvCFEM12 Is an Important Candidate Effector in F. verticillioides CFEM Family

**Figure 2:**
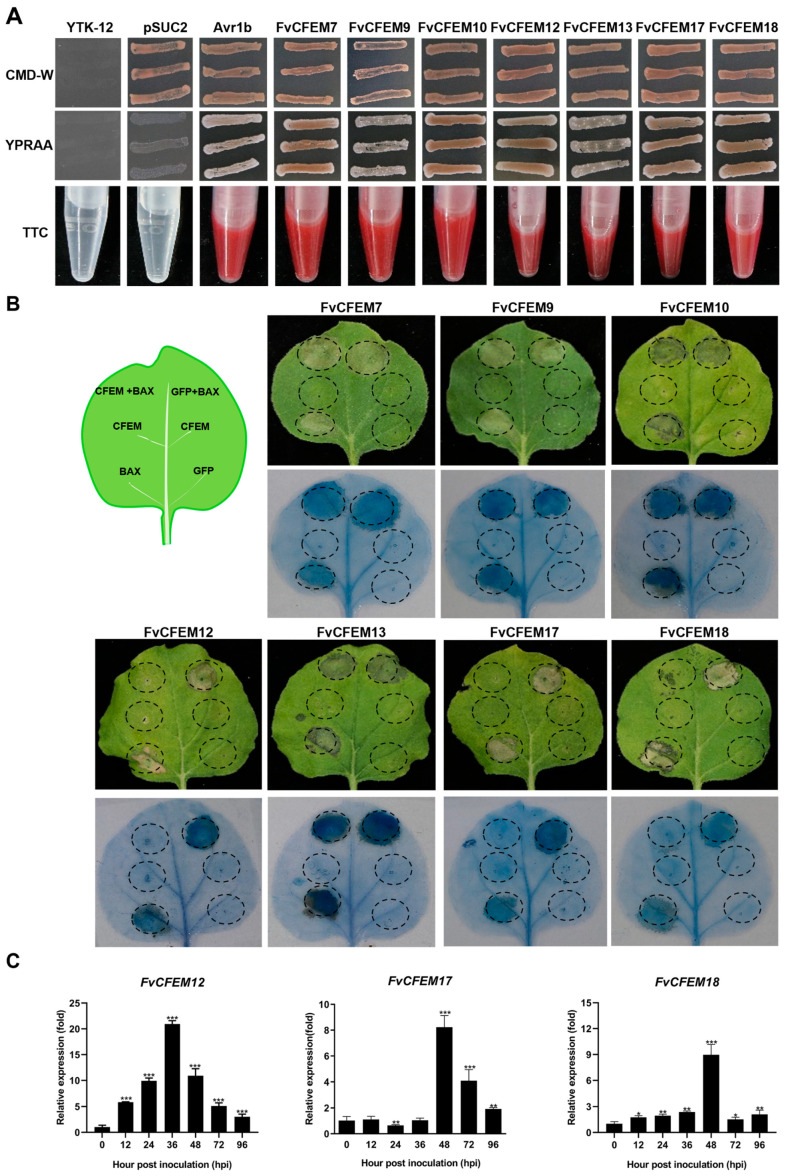
Identification of potential effectors among FvCFEM proteins. (**A**) Functional validation of the putative N-terminal signal peptide of FvCFEM proteins using a yeast signal trap assay. The putative signal peptide sequences of FvCFEMs were fused into the pSUC2 vector, and transformed into *S. cerevisiae* strain YTK12. Growth on CMD-W and YPRAA plates, as well as invertase activity in TTC reagent were assessed. Untransformed YTK12 and the YTK12 carrying the empty pSUC2 vector served as negative controls, while the signal peptide of the oomycete effector Avr1b was used as a positive control. (**B**) Transient expression of seven FvCFEM proteins in *N. benthamiana* leaves 24 h prior to infiltration with *A. tumefaciens* expressing Bax. Photographs were taken at 5 days post-inoculation (dpi), and dead cells were visualized by trypan blue staining. (**C**) Expression profile of FvCFEM12, FvCFEM 17, and FvCFEM 18 during *F. verticillioides* infection of maize. RT-qPCR analysis was performed on samples collected at 0, 12, 24, 36, 48, 72, and 96 h post-inoculation (hpi). *ZmUbiquitin* was used as an internal reference. Error bars represent the standard deviation of three biological replicates. Data are presented as means ± standard error of three biological replicates. *, ** and *** indicate significant differences at *p* < 0.05, *p* < 0.01 and *p* < 0.001, respectively, based on Student’s *t*-test.

Fungal pathogen effectors usually contain a signal peptide and lack transmembrane regions, playing crucial roles in pathogenicity [32]. Based on these criteria, seven FvCFEM proteins (FvCFEM7, 9, 10, 12, 13, 17, and 18) were predicted to possess a signal peptide without transmembrane regions. These proteins were considered as candidate effectors and selected for further investigation. To validate the functionality of the predicted signal peptides, we performed a yeast invertase secretion assay [33]. The signal peptides of the seven candidate effectors were successfully cloned into the pSUC2 vector and transformed into yeast strain YTK-12, which is unable to metabolize raffinose as a carbon source. Transformants were streaked onto CMD-W medium and YRPAA medium. Similar to the positive control (Avr1bSP), transformants containing the FvCFEM signal peptide grew on the YRPAA medium (Figure 2A). Furthermore, the enzyme activity of the secreted invertase was confirmed using 2,3,5-triphenyltetrazolium chloride (TTC). Transformants containing signal peptides from Avr1b and FvCFEMs resulted in a red color change in the reaction reagents. These results demonstrated that the predicted signal peptides of FvCFEM proteins are functional and capable of mediating protein secretion.

Some fungal effectors can induce or suppress plant cell death to facilitate infection and survival [34,35]. To investigate whether the candidate FvCFEM proteins modulate programmed cell death (PCD) in plants, we conducted transient expression assays in *N. benthamiana* using an *Agrobacterium*-mediated approach. Negative control (Green fluorescent protein, GFP) and positive control (BCL2-associated X, Bax) were included for comparison, and trypan blue staining was used to visualize cell death. The results revealed that none of the seven candidate FvCFEM proteins induced significant cell death; FvCFEM12, FvCFEM17, and FvCFEM18 were found to suppress Bax-induced PCD (Figure 2B). The expression of these proteins in *N. benthamiana* was confirmed by reverse transcription PCR (RT-PCR) (Figure S1). Based on these results, FvCFEM12, FvCFEM17, and FvCFEM18 were selected for further analysis.

To explore the expression patterns of FvCFEM12, FvCFEM17, and FvCFEM18 during *F. verticillioides* infection, we inoculated maize cultivar B73 with conidia from the wild-type strain FvLNF15-11 and performed quantitative real-time reverse transcription PCR (qRT-PCR) on infected stalk tissues. The results revealed distinct expression profiles for each gene. FvCFEM12 was induced as early as 6 h post-inoculation (hpi) and increased progressively, peaking at 36 hpi. In contrast, FvCFEM17 exhibited high expression levels during the later stages of infection, while FvCFEM18 showed a transient peak at 48 hpi, with low expression at other time points (Figure 2C). These findings suggest that FvCFEM12 may play a critical role during infection.

### 2.3. FvCFEM12 Suppresses Cell Death Induced by INF1 and P. syringae DC3000

**Figure 3:**
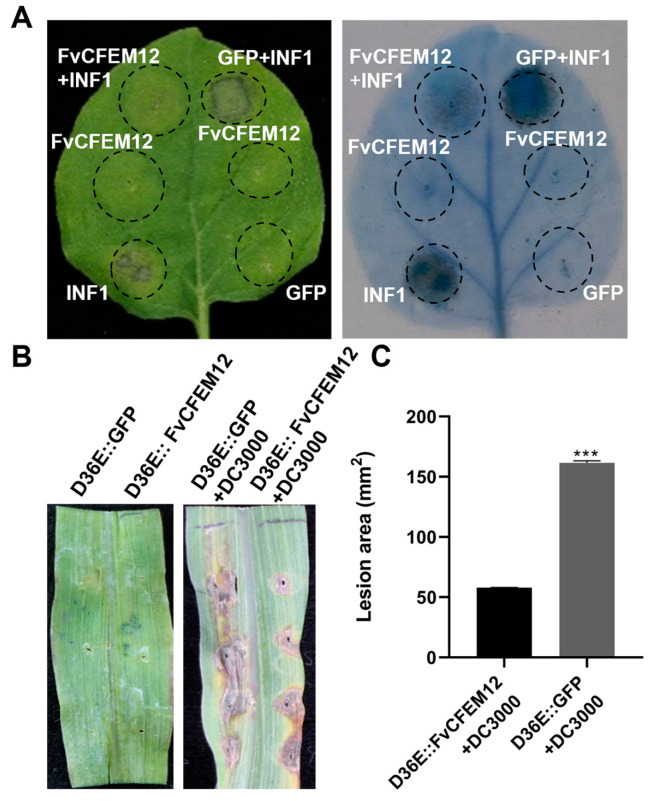
FvCFEM12 suppresses cell death triggered by INF1 and *P. syringae* DC3000. (**A**) FvCFEM12 was transiently expressed in *N. benthamiana* leaves 24 h prior to infiltration with *A. tumefaciens* expressing the *INF1* gene. Photographs were taken at 5 days post-inoculation (dpi), and lesion areas of leaves were visualized by trypan blue staining. (**B**) *P. syringae* strain D36E carrying FvCFEM12 was transiently expressed in maize leaves 24 h prior to infiltration with *P. syringae* strain DC3000. *P. syringae* strain D36E carrying GFP was used as a control. Photographs were taken at 5 dpi after inoculation with *P. syringae* strain DC3000. (**C**) Average lesion area on maize leaves was calculated from three independent biological replicates. Error bars represent the standard deviation (SD). Statistical analysis was determined using Student’s test (***, *p*-value < 0.001).

To determine whether FvCFEM12 can suppress cell death triggered by INF1, we transiently expressed FvCFEM12 in *N. benthamiana* leaves using *Agrobacterium*-mediated transformation. GFP was used as a negative control. Subsequently, 24 h after infiltration, the leaves were infiltrated with *A. tumefaciens* cells carrying the *INF1* gene. Cell death was significantly suppressed in regions expressing FvCFEM12, whereas no suppression was observed in GFP-expressing control leaves (Figure 3A). These results indicate that FvCFEM12 can inhibit INF1-induced cell death in *N. benthamiana*.

To further investigate the suppressive activity of FvCFEM12 in the host plant, we utilized a modified bacterial type III secretion system to deliver FvCFEM12 into maize [36]. FvCFEM12 and GFP were expressed in *P. syringae* strain D36E, which lacks all 36 known type III effectors (T3Es) and is non-pathogenic. When FvCFEM12 or GFP was injected alone, neither elicited significant cell death. However, five days after injection of *P. syringae* strain DC3000, regions expressing GFP exhibited extensive necrosis, whereas the region expressing FvCFEM12 showed a decrease in necrotic area (Figure 3B, C). These findings suggest that FvCFEM12 can suppress plant cell death triggered by both INF1 and *P. syringae* DC3000, further supporting its role as a suppressor of plant immune responses.

### 2.4. Transient Expression of FvCFEM12 Enhances Susceptibility to B.cinerea and Suppresses Plant Immunity

**Figure 4:**
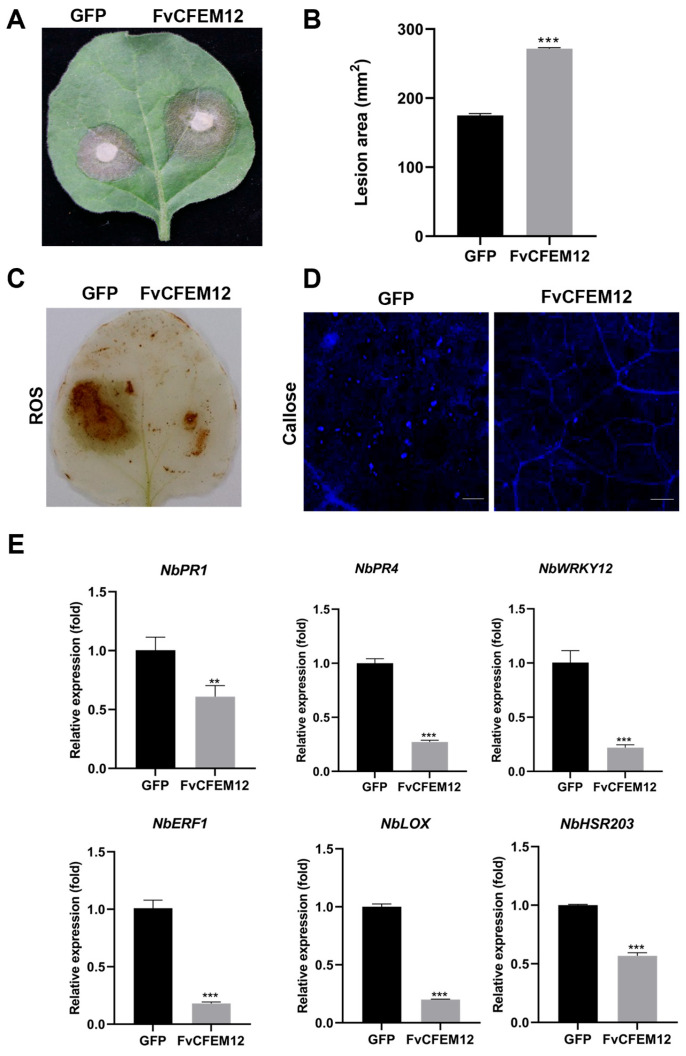
FvCFEM12 suppresses plant resistance responses and enhances susceptibility to *B. cinerea*. (**A**) *B. cinerea* infection assays in 4-week-old *N. benthamiana* leaves. The left side of the leaves was transiently expressed with *A. tumefaciens* carrying a GFP vector as a control, while the right side was transiently expressed with *A. tumefaciens* carrying the FvCFEM12 recombinant vector. Lesion phenotypes (**A**) and diameters (**B**) were examined at 2 days post-inoculation with *B. cinerea*. (**C**) Reactive oxygen species (ROS) accumulation and (**D**) callose deposition in *N. benthamiana* leaves transiently expressing FvCFEM12 were detected using 3,3’-diaminobenzidine (DAB) and aniline blue staining, respectively. Scale bar = 200 µm. (**E**) Relative expression levels of the defense-related genes (*NbRP1*, *NbPR4*, *NbWRKY12*, *NbERF1*, *NbLOX*, and *NbHSR203*) in *N. benthamiana* leaves infiltrated with *A. tumefaciens* carrying the FvCFEM12 recombinant vector and induced by *B. cinerea*. *A. tumefaciens* carrying the GFP vector was used as a control. *NbActin* served as an internal reference. Data are presented as means ± standard error of three biological replicates. ** and *** indicate significant differences at *p* < 0.01 and *p* < 0.001, respectively, based on Student’s *t*-test.

To investigate whether FvCFEM12 regulates plant immunity responses, we transiently expressed FvCFEM12 in *N. benthamiana* leaves, using GFP as a control on the opposite side of the same leaves. Two days after inoculation with *B. cinerea*, the lesion area in leaves expressing FvCFEM12 was significantly larger compared to the GFP-expressing control (Figure 4A,B). These results suggest that FvCFEM12 promotes the infection of *B. cinerea* in *N. benthamiana* plants.

Plant defense responses against pathogen infection are typically involved in ROS accumulation, callose deposition, and regulation of defense-related genes [37,38]. To further investigate the impact of FvCFEM12 on these defense responses, we measured ROS accumulation, callose deposition, and the expression of defense-related genes in *N. benthamiana* leaves inoculated with *B. cinerea*. ROS accumulation and callose deposition were significantly reduced in leaves expressing FvCFEM12 compared to the GFP control (Figure 4C,D). Additionally, the expression levels of six defense-related genes, including *NbPR1*, *NbPR4*, *NbWRKY12*, *NbERF1*, *NbLOX* and *NbHSR203*, were markedly downregulated in FvCFEM12-expressing leaves (Figure 4E). Taken together, these findings demonstrate that FvCFEM12 can suppress plant immune responses in *N. benthamiana* plants.

### 2.5. CFEM Domain Required for FvCFEM12 Cell Death Suppression, Independent of Subcellular Localization

**Figure 5:**
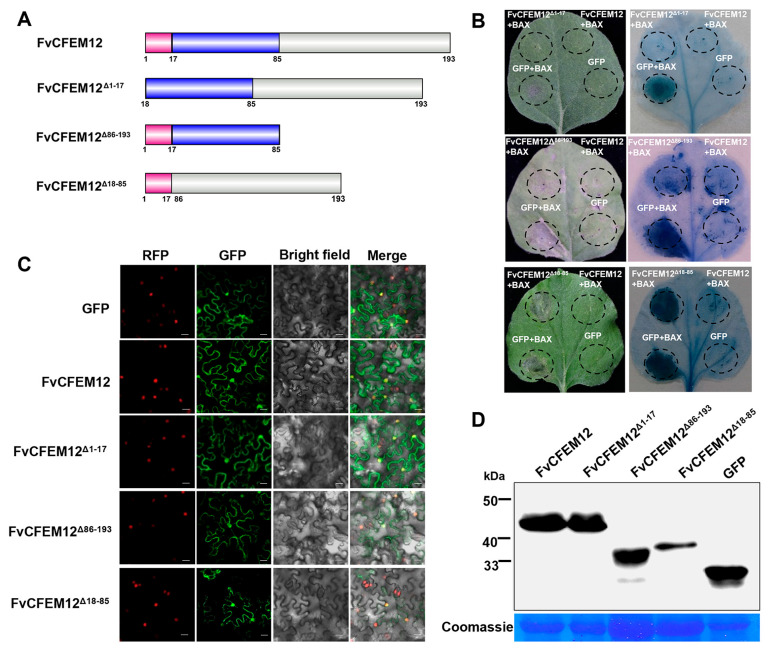
FvCFEM12 suppress Bax-induced cell death dependent on the CFEM domain. (**A**) Schematic representation of each mutant construct of FvCFEM12 generated using the IBS 2.0 website. The first construct, FvCFEM12, contains the full-length sequence of FvCFEM12. The second construct, FvCFEM12^Δ1−17^, lacks the signal peptide domain. The third construct, FvCFEM12^Δ86−193^, lacks the C-terminal sequence. The fourth construct, FvCFEM12^Δ17−185^, lacks the CFEM domain. Pink, blue, and grey blocks represent the signal peptide, CFEM domain, and unidentified sequence, respectively. (**B**) Different FvCFEM12 mutants were transiently expressed in *N. benthamiana* leaves 24 h prior to infiltration with *A. tumefaciens* carrying the *Bax* gene. Photographs were taken at 5 days post-inoculation (dpi), and lesion areas were visualized by trypan blue staining. (**C**) Subcellular localization of FvCFEM12 and its mutants fused with a C-terminal GFP in transgenic *N. benthamiana* leaves expressing H2B-RFP. GFP and RFP fluorescence were observed at 2 days post-agroinfiltration. Scale bar = 20 µm. (**D**) Western blot analysis of proteins extracted from *N. benthamiana* leaves transiently expressing FvCFEM12 and its mutants fused with a GFP tag.

To investigate the functional roles of the signal peptide and the conserved CFEM domain in FvCFEM12-mediated suppression of cell death, we generated a series of deletion mutants (Figure 5A). The first mutant, FvCFEM12^Δ1−17^, lacked the signal peptide; the second mutant, FvCFEM12^Δ86−193^, lacked the C-terminal region; and the third mutant, FvCFEM12^Δ18−85^, lacked the CFEM domain. Transient expression assays in *N. benthamiana* revealed that both FvCFEM12^Δ1−17^ and FvCFEM12^Δ86−193^ retained the ability to suppress cell death, similar to the full-length FvCFEM12. In contrast, the FvCFEM12^Δ18−85^ mutant, which lacks the CFEM domain, completely lost its cell death suppression activity (Figure 5B). These results demonstrate that the CFEM domain is essential for FvCFEM12’s function in suppressing cell death, while the signal peptide and C-terminal region are dispensable for this activity.

To further explore the subcellular localization of FvCFEM12, we transiently expressed FvCFEM12-GFP fusion protein in *N. benthamiana* leaves. Fluorescence microscopy revealed that the green fluorescence signal from FvCFEM12-GFP was distributed throughout the membrane and co-localized with the red fluorescent signal of the nuclear marker H2B-RFP, indicating that FvCFEM12 localizes to both the membrane and nucleus (Figure 5C). Furthermore, a similar localization pattern was observed for the deletion mutants FvCFEM12^Δ1−17^-GFP, FvCFEM12^Δ86−193^-GFP, and FvCFEM12^Δ18−85^-GFP. Expression of the GFP fusion proteins was verified by Western blot (Figure 5D). These results suggest that FvCFEM12 localized to both the membrane and nucleus in tobacco epidermal cells.

### 2.6. FvCFEM12 Is Involved in Phenotype Development and Cell Wall Stress Responses

**Figure 6:**
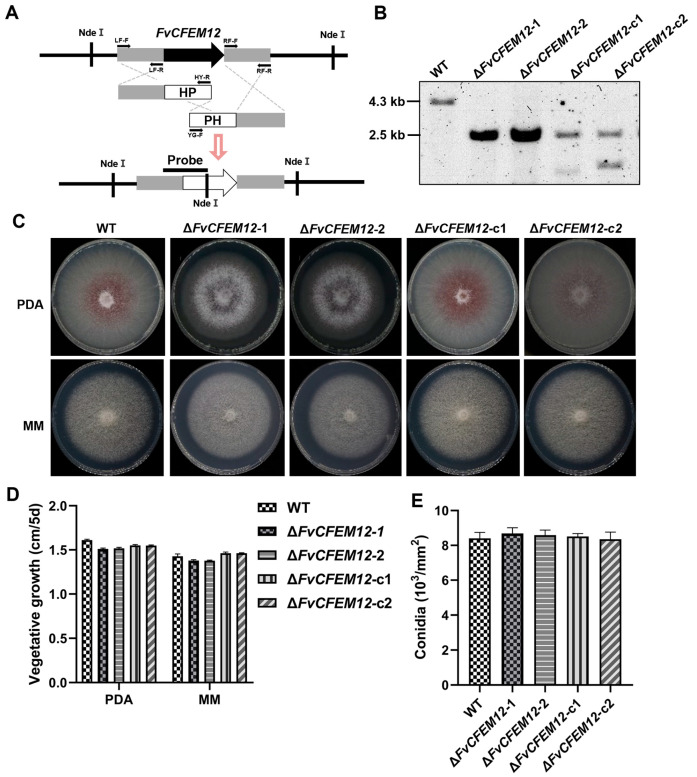
FvCFEM12 deletion affects colony phenotype, but not conidial germination. (**A**) Schematic strategy of the FvCFEM12 deletion based on the split-marker recombination method. NdeI digestion sites and the probe used for Southern hybridization are indicated. HP and PH represent different regions of the hygromycin B phosphotransferase (*hph*) marker gene cassette. Primers LF-F, LF-R, RF-F, RF-R, HY-F, and YG-F were used for gene replacement. (**B**) Southern blot analysis of genomic DNA from the wild-type FvLNF15-11 (lane 1), deletion mutants Δ*FvCFEM12*-1 (lane 2) and Δ*FvCFEM12*-2 (lane 3), and complemented strains Δ*FvCFEM12*-c1 (lane 4) and Δ*FvCFEM12*-c2 (lane 5). (**C**) Mycelial growth phenotypes and (**D**) Colony diameters of the wild-type FvLNF15-11, deletion mutants Δ*FvCFEM12*-1 and Δ*FvCFEM12*-2, and complemented strains Δ*FvCFEM12*-c1 and Δ*FvCFEM12*-c2 on PDA and MM agar plates. Photographs were taken at 5 days post-inoculation (dpi). (**E**) Quantification of conidia produced by the wild-type FvLNF15-11, deletion mutants Δ*FvCFEM12*-1 and Δ*FvCFEM12*-2, and complemented strains Δ*FvCFEM12*-c1 and Δ*FvCFEM12*-c2 on PDA agar plates. Data are presented as means ± standard error of three biological replicates. Data are presented as mean ± standard error from three biological replicates, with no statistically significant differences observed between wild-type and deletion mutants using Student’s *t*-test.

To investigate the biological function of FvCFEM12, we generated two deletion mutants (Δ*FvCFEM12*-1 and Δ*FvCFEM12*-2) using a split-marker approach, in which the FvCFEM12 gene was replaced with a hygromycin resistance (*hph*) cassette (Figure 6A). Complementary strains (Δ*FvCFEM12*-c1 and Δ*FvCFEM12*-c2) were created by reintroducing a functional copy of FvCFEM12 into the respective mutant strains. Southern blot analysis confirmed the successful generation of the mutants and complemented strains (Figure 6B).

To assess the role of FvCFEM12 in fungal growth and development, the wild-type (WT) strain, deletion mutants (Δ*FvCFEM12*-1 and Δ*FvCFEM12*-2), and complemented strains (Δ*FvCFEM12*-c1 and Δ*FvCFEM12*-c2) were cultured on Potato Dextrose Agar (PDA) and minimal medium (MM). Compared to the WT and complemented strains, the deletion mutants exhibited reduced pigment and loss of aerial hyphae at colony margin (Figure 6C,D). However, no significant differences were observed in conidia production, indicating that FvCFEM12 is involved in colony morphology but does not affect conidiation (Figure 6E).

**Figure 7:**
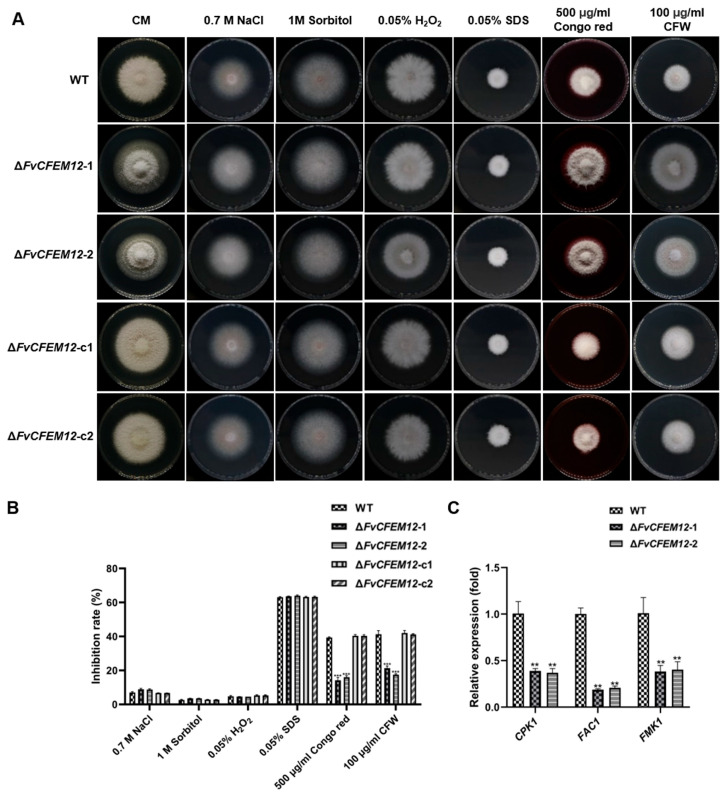
FvCFEM12 deletion alters tolerance to cell wall stress. (**A**) Mycelial growth phenotypes of the wild type FvLNF15-11, the deletion mutant Δ*FvCFEM12*-1 and Δ*FvCFEM12*-2 and complemented strains Δ*FvCFEM12*-c1 and Δ*FvCFEM12*-c12 on CM plates supplemented with 0.7 M NaCl, 1 M sorbitol, 0.05% H_2_O_2_, 0.05% SDS, 100 μg/mL CFW, and 500 μg/mL Congo red. Photographs were taken at 5 days post-inoculation (dpi). (**B**) Inhibition of mycelial radial growth in response to cell wall stressors. (**C**) Relative expression levels of the genes related to the cAMP-PKA and MAPK pathways in the wild type FvLNF15-11, deletion mutant Δ*FvCFEM12*-1 and Δ*FvCFEM12*-2 and complemented strains Δ*FvCFEM12*-c1 and Δ*FvCFEM12*-c2. *FvTubulin* was used as an internal reference. Data are presented as means ± standard error of three biological replicates. ** and *** indicate significant differences at *p* < 0.01 and *p* < 0.001, respectively, between the wild-type and deletion mutants, as determined by Student’s *t*-test.

Given the observed phenotypic changes, we hypothesized that FvCFEM12 might play a role in stress responses. To test this, we evaluated the growth of the WT, mutant, and complemented strains in CM medium supplemented with various stressors, including 0.7 M NaCl, 1 M sorbitol, 0.05% H_2_O_2_, 0.05% SDS, 100 μg/mL calcofluor white (CFW), and 500 μg/mL Congo Red. While no significant differences were observed in sensitivity to NaCl, sorbitol, H_2_O_2_, or SDS, the Δ*FvCFEM12* mutants exhibited reduced sensitivity to cell wall stressors (CFW and Congo Red) compared to the WT and complemented strains (Figure 7A, B).

The PKA and MAPK signaling pathways are known to regulate adaptive responses to environmental stresses [39]. To explore whether FvCFEM12 influences these pathways, we analyzed the expression of key genes (*CPK1*, *FAC1*, and *FMK1*) using qRT-PCR. The Δ*FvCFEM12* mutants showed significantly reduced expression of these genes compared to the WT strain (Figure 7C), indicating that FvCFEM12 may modulate stress responses through the PKA and MAPK pathways. Collectively, these results demonstrate that FvCFEM12 is involved in colony morphology and cell wall stress responses, potentially by regulating the PKA and MAPK signaling pathways.

### 2.7. FvCFEM12 Is Required for the Full Virulence of F. verticillioides

**Figure 8:**
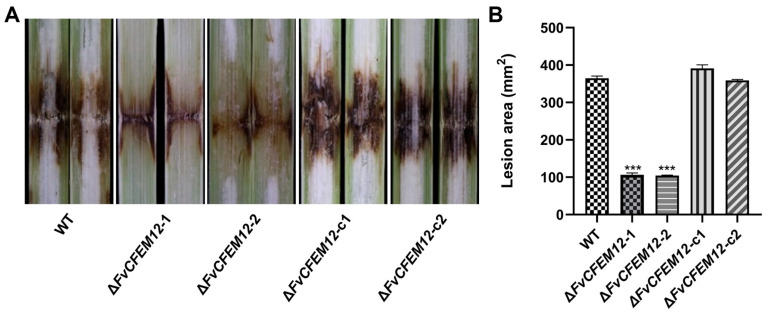
Pathogenicity assays of the wild type FvLNF15-11, deletion mutant Δ*FvCFEM12*-1 and Δ*FvCFEM12*-2 and complemented strains Δ*FvCFEM12*-c1 and Δ*FvCFEM12*-c2 on susceptible maize cultivar B73. (**A**) Phenotypes of maize stalks inoculated with the wild-type (WT), FvCFEM12 deletion mutants (Δ*FvCFEM12*-1 and Δ*FvCFEM12*-2), and complemented strains (Δ*FvCFEM12*-c1 and Δ*FvCFEM12*-c2) at 7 days post-inoculation. (**B**) Lesion areas on maize stalks were quantified using ImageJ1.46. Data are presented as means ± standard error of three biological replicates. *** indicates significant differences at *p* < 0.001 between the wild-type and deletion mutants, as determined by Student’s *t*-test.

To further investigate the role of FvCFEM12 in pathogenicity, we inoculated the susceptible maize cultivar B73 with the wild-type (WT) strain, FvCFEM12 deletion mutants (Δ*FvCFEM12*-1 and Δ*FvCFEM12*-2), and complemented strains (Δ*FvCFEM12*-c1 and Δ*FvCFEM12*-c2). Lesion areas on infected maize stalks were measured at 7 days post-inoculation. The Δ*FvCFEM12*-1 and Δ*FvCFEM12*-2 mutants exhibited significantly smaller lesion areas compared to the WT strain, while the complemented strains (Δ*FvCFEM12*-c1 and Δ*FvCFEM12*-c2) displayed lesion areas similar to those of the WT (Figure 8A,B). These results demonstrate that FvCFEM12 is essential for the full virulence of *F. verticillioides* during maize infection.

### 2.8. FvCFEM12 Interacts with Wall-Associated Receptor Kinase ZmWAK17

**Figure 9:**
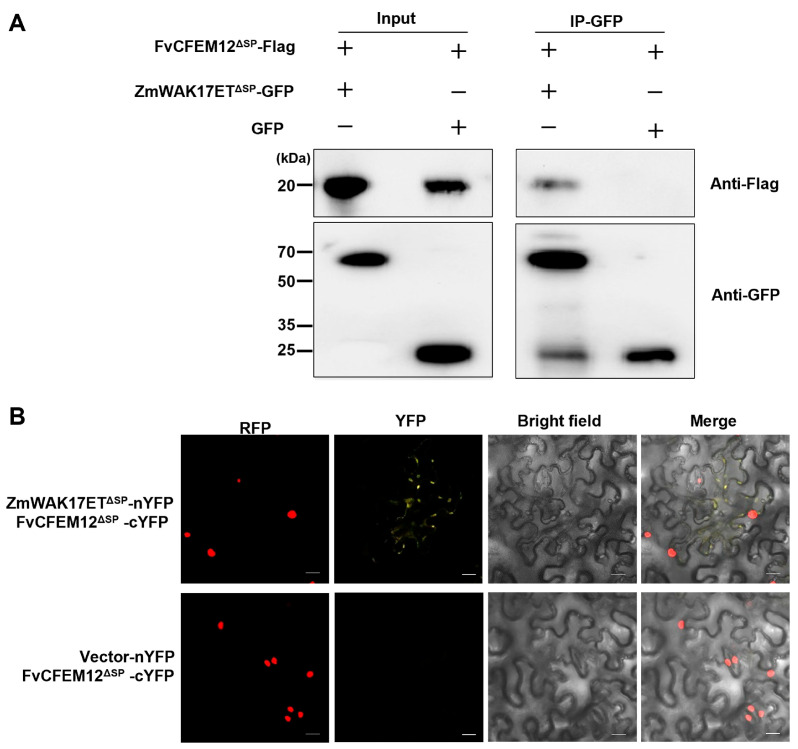
FvCFEM12 interacts with ZmWAK17ET. (**A**) Interaction between FvCFEM12-Flag and ZmWAK17ET-GFP was confirmed by co-immunoprecipitation (Co-IP). Total protein (Input) extracted from *N. benthamiana* leaves expressing FvCFEM12-Flag and ZmWAK17ET-GFP (or FvCFEM12-Flag and GFP) was subjected to SDS-PAGE, and immunoblots were incubated with anti-Flag and anti-GFP antibodies (Input panel). GFP-Trap beads were used to enrich ZmWAK17ET-GFP and GFP. FvCFEM12-Flag was detected in the ZmWAK17ET-GFP immunoprecipitate but not with GFP. (**B**) BiFC visualization of the interaction between FvCFEM12-cYFP and ZmWAK17ET-nYFP in transgenic *N. benthamiana* leaves expressing RFP-H2B. Co-expression of FvCFEM12-cYFP and vector-nYFP served as a control. YFP signals were observed using a confocal microscope at 2 days post-agroinfiltration. Scale bar = 20 µm.

Previous studies have shown that CFEM proteins in *F. graminearum* can interact with extracellular binding proteins ZmWAK17ET, which is an alternative splicing isoform of ZmWAK17 [40]. Interestingly, FvCFEM12 shares 50.62% amino acid sequence similarity with FgCFEM1, suggesting a potential functional conservation. To investigate whether FvCFEM12 interacts with ZmWAK17ET, we performed co-immunoprecipitation (Co-IP) assays. ZmWAK17ET was fused to GFP and FvCFEM12 was tagged with a Flag epitope, followed by co-expression in *N. benthamiana* leaves. Immunoprecipitation of ZmWAK17ET-GFP using anti-GFP antibodies specifically pulled down FvCFEM12-Flag, as detected by anti-Flag immunoblotting, whereas no signal was observed in the GFP control (Figure 9A), demonstrating a specific interaction between FvCFEM12 and ZmWAK17ET. To further validate this interaction, we conducted a bimolecular fluorescence complementation (BiFC) assay. Recombinant plasmids encoding pUC-SPYCE-FvCFEM12 and pUC-SPYNE-ZmWAK17ET were constructed and transiently co-expressed in *N. benthamiana* leaves via *A. tumefaciens*-mediated transformation. YFP fluorescence signals were observed at the plasma membrane microdomains, indicating a direct interaction between FvCFEM12 and ZmWAK17ET (Figure 9B). These results collectively demonstrate that FvCFEM12 physically interacts with ZmWAK17ET, suggesting a potential role in modulating plant immunity.

### 2.9. Silencing ZmWAK17 in Maize Reduce Resistance to F. verticillioides

**Figure 10:**
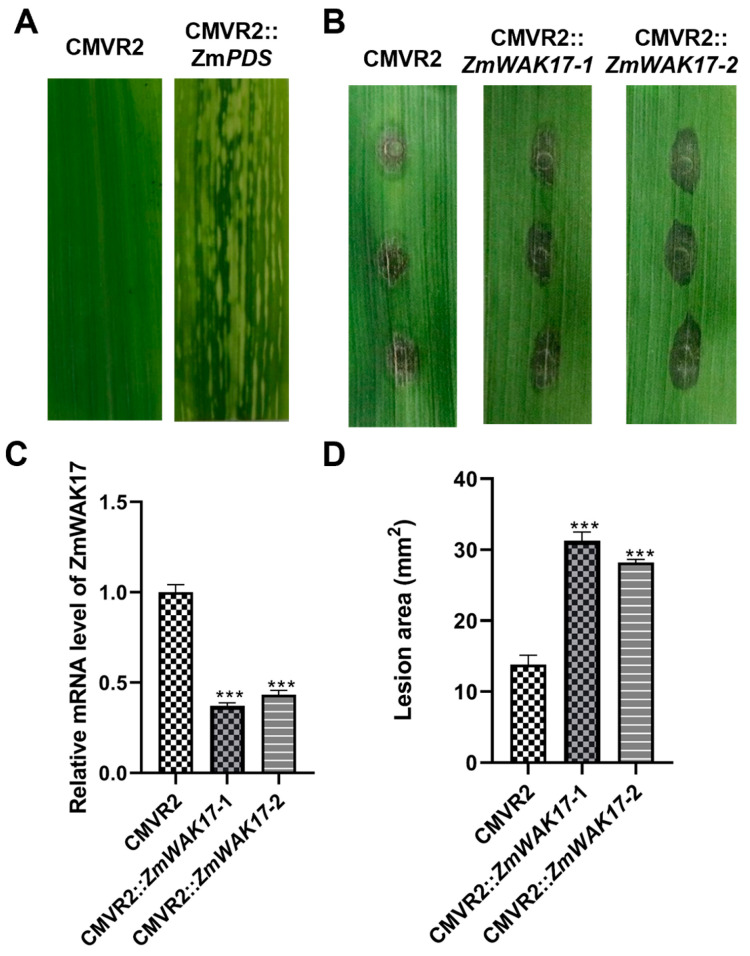
Silencing and functional assays of ZmWAK17 in maize resistance against *F. verticillioides*. (**A**) Leaf phenotypes of maize leaves expressing pCMVZ22bN81 (CMVR2) and CMVR2::*ZmPDS* at 12 days post-agroinfiltration. (**B**) Transcript levels of ZmWAK17 in ZmWAK17-silenced maize leaves. (**C**) Phenotypes and (**D**) lesion area of ZmWAK17 silenced maize leaves infected with *F. verticillioides* at 5 days post-inoculation (dpi). Empty vector CMVR2 silenced maize leaves were used as a control. Data are presented as means ± standard error of three biological replicates. *** indicates significant differences at *p* < 0.001 between CMVR2 and CMVR2::ZmWAK17-1/2, as determined by Student’s *t*-test.

To investigate the role of ZmWAK17 in maize resistance against *F. verticillioides*, we employed a Cucumber Mosaic Virus (CMV)-mediated virus-induced gene silencing (VIGS) system to downregulate ZmWAK17 expression in maize cultivar B73. Phytoene desaturase (PDS) was used as a positive control for silencing efficiency, while an empty vector (pCMVZ22bN81) served as a negative control. Successful silencing was confirmed by the appearance of photo-bleaching phenotypes in ZmIspH-silenced plants (Figure 10A). Following *F. verticillioides* inoculation, leaves were collected to assess ZmWAK17 expression levels using qRT-PCR. The results showed a significant ~65% reduction in ZmWAK17 expression in silenced plants compared to the control group (Figure 10B). At 5 days post-inoculation, ZmWAK17-silenced plants exhibited increased susceptibility to *F. verticillioides* infection, as evidenced by larger lesion areas compared to control plants (Figure 10 C,D). These findings demonstrate that ZmWAK17 plays a critical role in maize resistance against *F. verticillioides*.

## 3. Discussion

CFEM proteins are uniquely found in fungi, and an increasing number of studies have highlighted their roles as effectors in plant pathogenic fungi. Effectors typically possess signal peptides, lack transmembrane regions, and can modulate plant cell death. These characteristics make it suitable for rapid screening of candidate effectors. Previous studies suggest that some CFEM proteins can suppress plant cell death [25,41], while only a few proteins, such as PstCFEM1 and BcCFEM1, have been reported to induce cell death or chlorosis in *N benthamiana* [27,41]. In this study, we identified FvCFEM12 as a CFEM protein capable of suppressing cell death induced by BAX, INF1 in *N. benthamiana*. Furthermore, we demonstrated that FvCFEM12 also inhibits cell death induced by *P. syringae* strain DC3000 on the host plant (Figure 3B and Figure 5). These findings suggest that FvCFEM12 functions as a potential effector protein.

CFEM effectors are known to play diverse roles in fungal growth and development, with notable functional variations across different fungal species. While certain CFEM effectors, like CfEC12 and FgCFEM1, have no observable impact on fungal morphology, others significantly influence spore formation and development. For instance, PeCFEM5 and PeCFEM8 affect spore morphogenesis without altering colony morphology, whereas CgCFEM1 is crucial for conidial development and invasion structure formation in *Colletotrichum gloeosporioides*. In this study, we demonstrate that FvCFEM12 significantly alters fungal phenotype by reducing pigmentation and inhibiting aerial hyphae formation at colony edges. These observations align with recent studies indicating that certain CFEM effectors affect fungal morphology. In *Neostagonosporella sichuanensis*, *NsCFEM1* deletion mutants exhibited denser mycelium compared to the wild type, with reduced pigmentation and accelerated colony expansion. Similarly, in *C*. *gloeosporioides*, ΔCgCsa mutants displayed impaired radial growth and defective hyphal tip development, suggesting a crucial role for CFEM proteins in maintaining hyphal polarity. Collectively, these findings highlight the functional diversification of CFEM effectors in regulating fungal morphogenesis.

Glycosylphosphatidylinositol (GPI)-anchored proteins are typically localized to the outer leaflet of the plasma membrane via a GPI anchor and play critical roles in maintaining cell wall integrity and function in fungi [42,43]. For example, the GPI-anchored protein Gas1p in *S. cerevisiae* is essential for cell wall formation [44], while Dfg3 in *A. fumigatus* is involved in the association of galactomannan with the β-(1,3)-glucan–chitin core of the cell wall [45]. These findings are consistent with previous reports on CFEM proteins (e.g., Rbt5, Rbt51, Csa1, and BcCFEM1) that similarly contain GPI anchors and are implicated in the regulation of cell wall biosynthesis [27,46]. In this study, we identified two putative GPI-modification sites within FvCFEM12, and deletion mutants exhibited altered sensitivity to cell wall stressors, suggesting a potential role in cell wall integrity. Notably, the majority of characterized CFEM proteins appear to positively regulate cell wall biosynthesis, while FvCFEM12 seems to function as a negative regulator. This may be due to the fact that different CFEM family proteins may serve distinct roles in various aspects of cell wall biology.

Plant pathogens often secrete effectors to manipulate host immune responses and facilitate infection [47]. Several CFEM proteins including PstCFEM1 from *P. striiformis* f. sp. *tritici* and BcCFEM1 from *B. cinerea* have been reported to be implicated in virulence [27,28,29,40]. In this study, transient expression of FvCFEM12 in *N. benthamiana* leaves promoted the infection process of *B. cinerea* and suppressed plant defense responses, including ROS accumulation, callose deposition, and the induced expression of defense-related genes (Figure 7). Pathogenicity assays on maize stalks further confirmed that FvCFEM12 contributes to the virulence of *F. verticillioides* (Figure 8). These studies collectively suggest that CFEM proteins play diverse roles in promoting virulence across fungal pathogens, warranting further investigation into the functions of other CFEM family members *F. verticillioides*.

**Figure 11:**
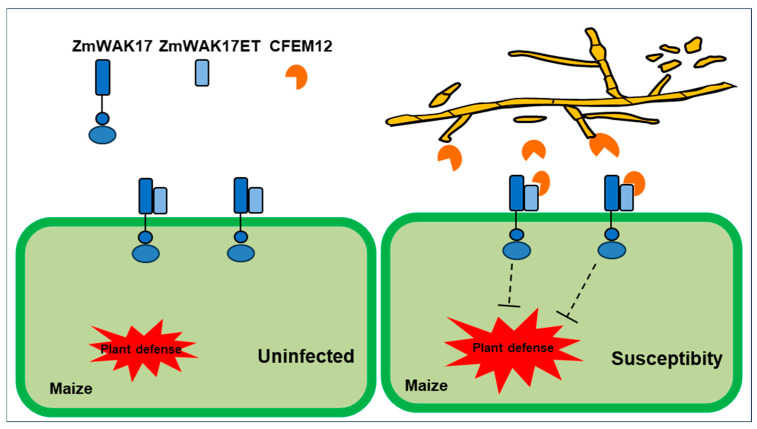
Proposed model of FvCFEM12 suppressing ZmWAK17-mediated plant immunity during *Fusarium verticillioides* infection. Under physiological conditions, ZmWAK17 acts as a pattern recognition receptor that activates basal immune responses through extracellular domain (ET)-mediated perception of pathogen-associated molecular patterns. During *F. verticillioides* infection, the fungal pathogen secretes the effector protein FvCFEM12, which interacts with ZmWAK17ET, thereby disrupting receptor-mediated signaling transduction. The effector–receptor interaction competitively inhibits ZmWAK17’s capacity to initiate defense-related pathways, ultimately facilitating fungal infection in maize tissues.

Despite the growing evidence of CFEM proteins’ roles in pathogenicity, the molecular mechanisms underlying their interactions with host plants remain poorly understood. In *F. graminearum*, CFEM proteins interact with the maize protein ZmWAK17ET to facilitate infection. Wall-associated kinases (WAKs), as members of the receptor-like kinase (RLK) superfamily, are involved in various biological processes, including growth, development, plant immunity, and abiotic stress responses [48,49]. Previous studies have demonstrated that ZmWAK17 confers resistance to *F. graminearum*; our findings reveal that ZmWAK17 also enhances resistance to *F. verticillioides*. This suggests a potential broad-spectrum resistance mechanism against multiple *Fusarium* species. Furthermore, we show that FvCFEM12 interacts with ZmWAK17ET, although the precise molecular mechanisms of this interaction and the downstream signaling pathways involved in ZmWAK17-mediated resistance remain to be elucidated. Based on our observations, we hypothesize that FvCFEM12 suppresses ZmWAK17-mediated resistance in maize, thereby promoting *F. verticillioides* infection (Figure 11). In summary, FvCFEM12 is identified as a crucial virulence CFEM effector in *F*. *verticillioides*. We propose that these CFEM effectors, which play significant roles in pathogenicity, could serve as potential targets for the management of plant fungal diseases.

## 4. Materials and Methods

### 4.1. Fungal Strains and Plant Materials

The *F. verticillioides* strain FvLNF15-11 was isolated from diseased maize stalks in Liaoning Province, China [50]. For colony morphology analysis, the wild-type strain FvLNF15-11, along with its mutants and complemented strains, were cultured on potato dextrose agar (PDA) and minimal medium (MM) plates. Stress assays were conducted by incubating all strains on complete medium (CM) plates supplemented with specific stressors, including 0.7 M NaCl, 1 M sorbitol, 0.05% hydrogen peroxide (H_2_O_2_), 0.05% sodium dodecyl sulfate (SDS), 100 μg/mL calcofluor white (CFW), and 500 μg/mL Congo Red (CR), for 5 days. The *B. cinerea* strain was cultured on PDA plates at 25 °C. *N. benthamiana* and maize cultivar B73 plants were grown in a greenhouse at 25 °C with a 16 h light and 8 h dark photoperiod.

### 4.2. Bioinformatics Identification of CFEM Proteins in F. verticillioides

To identify CFEM proteins in *F. verticillioides*, we employed a search strategy using the CFEM-containing protein ACI1 from M. oryzae as a query in the NCBI database (https://blast.ncbi.nlm.nih.gov/Blast.cgi, accessed on 18 January 2024). The candidate sequences retrieved from this search were subsequently analyzed for the presence of the CFEM domain using the Pfam tool available on the SMART platform (http://smart.embl-heidelberg.de/, accessed on 20 January 2024). For further characterization, the presence of N-terminal signal peptides was predicted using SignalP 5.0 (https://services.healthtech.dtu.dk/service.php?SignalP-5.0, accessed on 2 February 2024). Transmembrane regions within the CFEM proteins were identified using the TMHMM server (https://dtu.biolib.com/DeepTMHMM, accessed on 20 January 2024). Additionally, potential GPI modification sites were predicted using the GPI modification site prediction tool (https://mendel.imp.ac.at/gpi/fungi_server.html, accessed on 22 January 2024).

### 4.3. Phylogenetic Analysis and Multiple Sequence Alignment

The amino acid sequences of the 19 CFEM-containing proteins from *F. verticillioides* were aligned using ClustalW, and the consensus sequence was visualized using Jalview [51]. A phylogenetic tree was constructed using the neighbor-joining method based on these sequences in MEGA 7.0 [24]. The reliability of the tree nodes was assessed through non-parametric bootstrapping with 1000 pseudo-replicates. The phylogeny and domain architecture of each protein were visualized using iTOL (https://itol.embl.de/, accessed on 12 February 2024). The alignment results of the CFEM domains were analyzed using WebLogo (http://weblogo.berkeley.edu/logo.cgi, accessed on 15 February 2024).

### 4.4. Analysis of Chromosomal Distribution of CFEM Genes

To systematically investigate the genomic organization characteristics of FvCFEM effector genes in *F. verticillioides*, we performed physical mapping of all 19 identified FvCFEM genes onto their respective chromosomes using MG2C v2.1 on the website (http://mg2c.iask.in/mg2c_v2.1/index.html, accessed on 20 July 2024).

### 4.5. Yeast Signal Sequence Trap Assays

The functionality of the predicted signal peptides in the seven CFEM proteins was validated using a previously described method [52]. Each predicted signal peptide was fused in-frame to the pSUC2 vector, and transformed into the yeast strain YTK-12 using the Frozen-EZ Yeast Transformation II kit (Zymo Research, Irvine, CA, USA). Transformed yeast cells were cultured on CMD-W medium (0.67% yeast nitrogen base without amino acids, 0.075% tryptophan dropout supplement, 2% sucrose, 0.1% glucose, and 2% agar) and YPRAA medium (1% yeast extract, 2% peptone, 2% raffinose, and 2 μg/mL antimycin A).

Invertase enzymatic activity was further confirmed using 2,3,5-triphenyltetrazolium chloride (TTC) staining assays. Yeast transformants were incubated in sodium acetate buffer (pH 4.7, 100 mM sucrose) for 10 min at 37 °C, followed by centrifugation at 10,000 rpm for 1 min. The supernatant was mixed with an alkaline 1% TTC solution, and a colorimetric change after 5 min at room temperature indicated invertase activity.

### 4.6. Transient Expression of CFEM Proteins in N. benthamiana and Z. mays

The full-length open reading frames (ORFs) of FvCFEMs genes were amplified and cloned into the expression plasmid pCHF3. All recombinant plasmids were transformed into *A. tumefaciens* strain C58C1 and cultured in Luria–Bertani (LB) medium supplemented with 50 µg/mL spectinomycin. Cultures were grown overnight at 28 °C until reaching an optical density (OD_600_) of 0.5–1.0. The bacterial cells were then washed three times with the resuspended buffer (10 mM MgCl_2_, 10 mM MES, and 150 uM acetosyringon) and adjusted to an OD_600_ of 0.4. After incubation for 3 h at room temperature, the bacterial suspensions were infiltrated into leaves of 4-week-old *N. benthamiana* plants. To assess cell death suppression, *A. tumefaciens* carrying BAX or INF1 genes was infiltrated into the same sites 24 h later. For subcellular localization, FvCFEM12-GFP fusion proteins were transiently expressed in transgenic *N. benthamiana* plants expressing RFP-H2B [53]. Leaves were examined at 3 days post-inoculation using a ZEISS LSM980 confocal scanning microscope.

For transient expression in maize, the method was adapted from Tang et al. [54] and Liang et al. [55] with modifications. The FvCFEM12 and GFP were first cloned into the Gateway donor vector pDONR 221 by BP recombination, and then subcloned into the Gateway expression vector pEDV6 through LR recombination (Invitrogen, Carlsbad, CA, USA). The recombinant plasmids were transformed into *P. syringae* strain D36E by electroporation (2.5 kv/6 ms). Transformed acteria were cultured in King’s B medium supplemented with 30 µg/mL gentamicin for 24 h at 28 °C. Cells were harvested by centrifugation (4000× *g*, 5 min), washed three times with 10 mM MgCl_2_, and resuspended in the same solution. The OD_600_ was adjusted to 0.5 and incubated at room temperature for 3 h. Subsequently, the bacterial suspension was injected into leaves of two-leaf-stage maize plants. After 24 h, *P. syringae* strain DC3000 was infiltrated into the same sites at OD_600_ = 0.8 in 10 mM MgCl_2_. Plants were maintained under high humidity and darkness for 24 h before being returned to normal conditions. The lesion areas were photographed 5 days post-inoculation and quantified using ImageJ 1.46 software.

### 4.7. RT-qPCR Analysis

Total RNA was extracted using Trizol reagent (TransGen Biotech, Beijing, China). First-strand cDNA was synthesized from the extracted RNA using the PrimeScript RT reagent Kit (Takara, Dalian, China.). Quantitative real-time PCR (qPCR) was performed using the NovoStart SYBR qPCR SuperMix (Novoprotein, Suzhou, China) on a Real-Time PCR detection system. The relative expression levels of target genes were normalized to internal controls: *FvTubulin* for *F. verticillioides*, *NbActin* for *N. benthamiana*, and *ZmUbiquitin* for *Z. mays*. Gene expression was quantified using the 2^−∆∆Ct^ method.

### 4.8. Trypan Blue Staining

Infiltrated *N. benthamiana* leaves were collected 3 days post-inoculation. A trypan blue solution was prepared by dissolving 10 mL lactic acid, 10 mL glycerol, 10 g phenol, and 10 mg trypan blue in 10 mL distilled water, with the final volume adjusted to 40 mL by adding distilled water. Leaves were immersed in a 1:1 mixture of trypan blue solution and ethanol (40 mL total volume) and heated at 90 °C for 2 min. After boiling, the stained leaves were incubated overnight at room temperature. Excess stain was removed by washing the leaves several times with distilled water. To reduce background staining, leaves were destained in hydrated trichloroacetaldehyde until the background color was cleared.

### 4.9. Detection of Oxidative Burst and Callose Deposition

To induce defense responses, a mycelial plug (0.7 cm in diameter) of *B. cinerea* was taken from the edge of the colony and placed on the infiltrated area of *N. benthamiana* leaves. Reactive oxygen species (ROS) accumulation was detected using a 3,3’-diaminobenzidine (DAB) solution kit (Solarbio, Beijing, China). Leaf samples were immersed in DAB solution (1 mg/mL) and shaken at 50 rpm for 12 h. After staining, leaves were bleached in 96% ethanol and photographed.

Callose deposition was analyzed using an aniline blue staining method with minor modifications [56]. Samples were decolorized in absolute ethanol and then incubated in 150 mM phosphate buffer (pH 9.5) containing 0.1% aniline blue for 2 h in the dark. Stained samples were observed under a ZEISS LSM 980 confocal scanning microscope using an excitation filter of 405 nm and an emission filter of 485–515 nm.

### 4.10. F. verticillioides Gene Knockout and Complementation

Gene deletion constructs were generated using a split-marker recombination approach as previously described [57]. The upstream and downstream flanking regions of the FvCFEM12 gene were amplified using primer pairs LF-F/LF-R and RF-F/RF-R, respectively. Different regions of the hygromycin B phosphotransferase (*hph*) gene were amplified using primers HYG-F/HY-R and YG-F/HYG-R. Overlapping PCR products, amplified with primer pairs LF-F and RF-R, were introduced into protoplasts of the wild-type strain. Protoplast generation and transformation were performed according to the method described by Ridenour et al. [58].

For complementation assays, the ORF of FvCFEM12, including its native promoter, was co-transformed with linearized pFL2 into the yeast strain XK12 to generate the complementation plasmid, as described by Jiang et al. [59].The complementation plasmid was then transformed into protoplasts of the FvCFEM12 deletion mutant to obtain the complemented strain.

### 4.11. Pathogenicity Assay

The pathogenicity of fungal strains on maize stalks was assessed as previously described [60]. Briefly, fungal strains were cultured on PDA medium at 25 °C for 5 days. Conidia were harvested by washing the fungal culture with sterile water and adjusted to a concentration of 1 × 10⁶ conidia/mL. At the 10-leaf stage, 100 µL of the conidial suspension was injected into maize stalks. Disease symptoms were photographed at 7 days post-inoculation, and lesion areas were quantified using ImageJ software.

### 4.12. Co-IP Assay

Recombinant plasmids pCHF3-FvCFEM12-Flag and pCHF3-ZmWAK17ET-GFP were transformed into *A. tumefaciens* strain C58C1 and co-infiltrated into *N. benthamiana* leaves. Two days post-infiltration, total protein was extracted from the leaves using lysis buffer (100 mM Tris-Cl, pH 7.5; 150 mM NaCl; 1% Triton X-100; 0.5 mM EDTA, pH 8.0; 0.1% SDS; 10 mM dithiothreitol; and protease inhibitor cocktail). GFP-Trap beads (Chromotek, Martinsried, Germany) were used to enrich GFP-tagged proteins. The beads were washed twice with wash buffer (100 mM Tris-HCl, pH 7.5; 150 mM NaCl; 0.05% Nonidet™ P40 Substitute; and 0.5 mM EDTA). Bound proteins were eluted using 200 mM glycine and analyzed by Western blot.

### 4.13. BiFC Assay

The FvCFEM12 gene was cloned into the vector pUC-SPYCE, and the ZmWAK17ET gene was cloned into the vector pUC-SPYNE. Recombinant plasmids were transformed into *A. tumefaciens* strain GV3101 and co-infiltrated into transgenic *N. benthamiana* plants expressing RFP-H2B. YFP and RFP fluorescence in infiltrated leaves was observed using a confocal laser microscope 48 to 72 h post-infiltration.

### 4.14. Virus-Induced Gene Silencing (VIGS) Assay in Maize

The CMV-mediated virus-induced gene silencing (CMV-VIGS) approach was implemented as previously described by Li H [61]. *ZmPDS* (phytoene desaturase gene) was used as the positive control for photobleaching phenotype validation. Gene-specific fragments (300 bp) targeting *ZmWAK17* (amplified with primers WAK17-VIGS-F/R, Supplementary Table S1) were cloned into the pCMVZ22bN81 vector through Kpn I/Xba I restriction sites and electroporated into *A. tumefaciens* strain GV3101. Agrobacterium cells harboring CMV RNAs 1, 2, and 3 constructs or their derivatives were equally mixed and infiltrated into *N. benthamiana* leaves. After four days, *N. benthamiana* leaves were homogenized in a PBS buffer. The homogenized leaf extract was then rub-inoculated onto the second maize leaves pre-dusted with 400-mesh carborundum. Following inoculation, the plants were kept in the dark for two days. After that, the plants were transferred to a growth room with a temperature of 25 °C and 16 h light/8 h dark cycle. The conidia suspension of *F. verticillioides* was adjusted to 1 × 10^6^ condia/mL in 0.02% Tween 20 solution. Silenced maize leaves were punched using a tissue puncher and inoculated with 10 ul conidia suspension. Disease symptoms on the maize leaves were photographed at 5 days post-inoculation and lesions were measured by Image J.

## 5. Conclusions

In summary, our study identified 19 CFEM proteins in the *F. verticillioides* genome, with FvCFEM12 emerging as a key candidate effector. We demonstrated that FvCFEM12 significantly contributes to fungal virulence and plays a key role in suppressing plant immunity. Additionally, FvCFEM12 was found to influence colony morphology and participate in cell wall synthesis. Importantly, we discovered that FvCFEM12 interacts with the maize protein ZmWAK17ET. Collectively, these findings provide valuable insights into the biological functions of CFEM proteins in *F. verticillioides* and their potential roles in fungal pathogenicity and host–pathogen interactions.

## Figures and Tables

**Table 1:** Identification of the Common Fungal Extracellular Membrane (CFEM) protein family in *F. verticillioides*.

Name	ID	SP ^a^	TM ^b^	Amino Acid	Position of CFEM Domain (aa)	GPI-Anchored
FvCFEM1	FVEG_16152	1–17	7	454	24–88	
FvCFEM2	FVEG_07166	1–20	6	460	34–98	
FvCFEM3	FVEG_13472	1–19	7	429	25–88	
FvCFEM4	FVEG_08756	1–19	6	431	27–88	
FvCFEM5	FVEG_17608		7	419	12–75	
FvCFEM6	FVEG_10583	1–22	7	442	23–86	
FvCFEM7	FVEG_04843	1–17	0	314	19–88	A^291^/A^314^
FvCFEM8	FVEG_06284		1	313	10–82	
FvCFEM9	FVEG_01052	1–17	0	154	18–85	N^133^/A^134^
FvCFEM10	FVEG_02239	1–15	0	196	17–84	S^166^/S^167^
FvCFEM11	FVEG_13696	1–18	7	423	20–84	
FvCFEM12	FVEG_07535	1–16	0	193	18–85	G^168^/S^173^
FvCFEM13	FVEG_08884	1–18	0	179	33–94	
FvCFEM14	FVEG_13561		7	590	173–231	
FvCFEM15	FVEG_17202	1–21	6	415	38–97	
FvCFEM16	FVEG_02591	1–21	1	306	23–82	
FvCFEM17	FVEG_08871	1–18	0	95	31–91	
FvCFEM18	FVEG_07573	1–19	0	245	21–84	D^219^
FvCFEM19	FVEG_02431		0	853	633–695	A^773^/G^774^

^a^ Cleavage site of signal peptide (SP) in the FvCFEM proteins. ^b^ Number of transmembrane domain (TM).

## Data Availability

The original contributions presented in the study are included in the article. Further inquiries can be directed to the corresponding author.

## Supplementary Materials

The following Supporting Information can be downloaded at: https://www.mdpi.com/article/10.3390/ijms26094369/s1.
