# Supplementary figures and images for: A Novel CFEM Effector in Fusarium verticillioides Required for Virulence Involved in Plant Immunity Suppression and Fungal Cell Wall Integrity
Source: Int J Mol Sci. 2025 May 4;26(9):4369. doi: 10.3390/ijms26094369 (PMC12072874; doi:10.3390/ijms26094369)

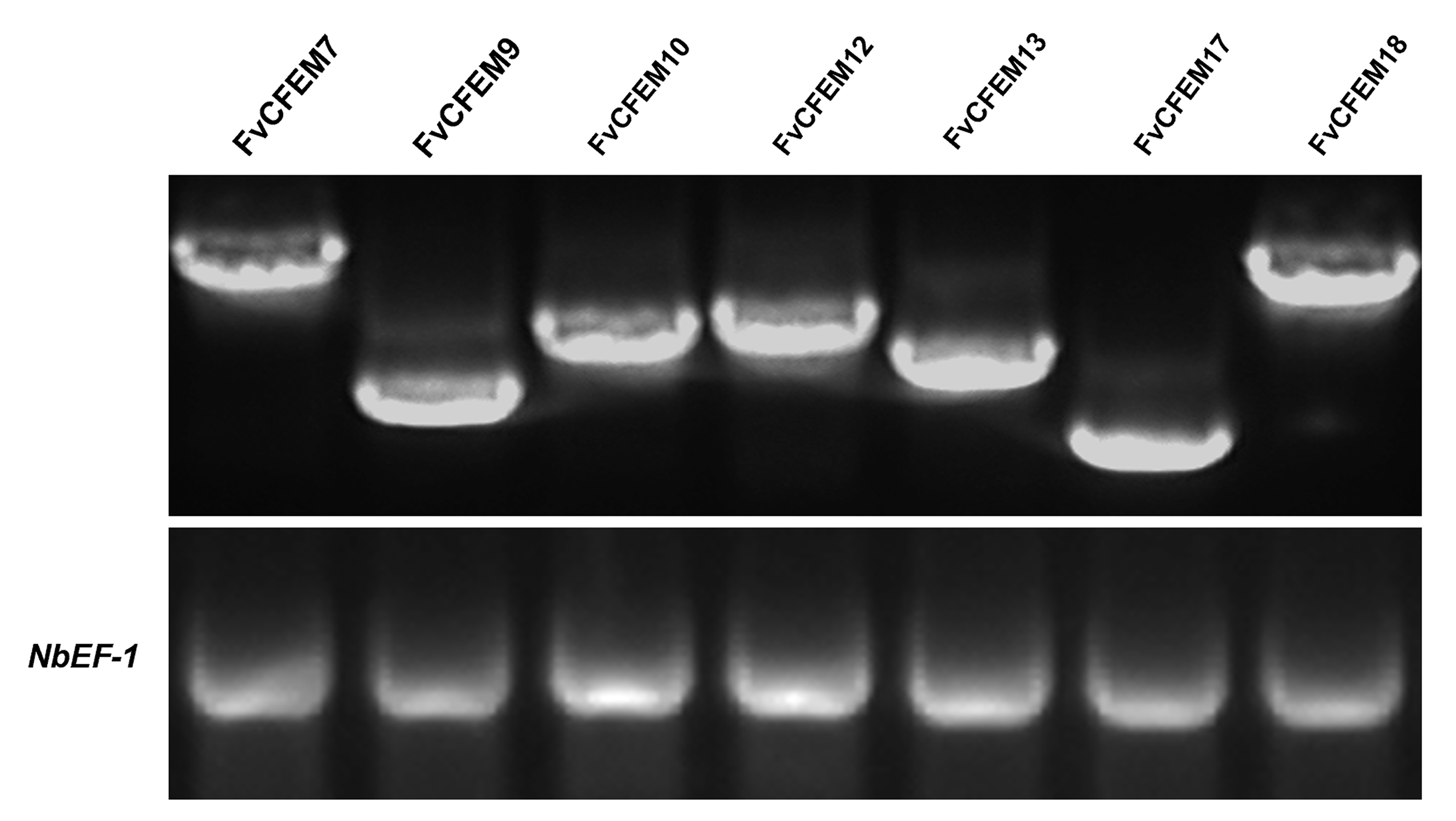

Supplement: Supplementary file 1 [file ijms-26-04369-s001.zip › Supplementary files/Fig.S1.tif]
